# Risk of suicide in households threatened with eviction: the role of banks and social support

**DOI:** 10.1186/s12889-019-7548-9

**Published:** 2019-09-11

**Authors:** Inmaculada Mateo-Rodríguez, Laura Miccoli, Antonio Daponte-Codina, Julia Bolívar-Muñoz, Cecilia Escudero-Espinosa, M. Carmen Fernández-Santaella, Jaime Vila-Castellar, Humbelina Robles-Ortega, José Luis Mata-Martín, Mariola Bernal-Solano

**Affiliations:** 1 CIBER Epidemiology and Public Health (CIBERESP), Instituto de Salud Carlos III, Madrid, Spain; 2 Andalusian School of Public Health (EASP), Granada, Spain; 3 Institute of Biomedicine Research (IBIS) Granada, Granada, Spain; 4 Andalusian Observatory on Environment and Health (OSMAN), Granada, Spain; 5 Mind, Brain, and Behavior Research Center (CIMCYC), University of Granada, Granada, Spain; 6 Observatorio de Salud y Medio Ambiente de Andalucía, Escuela Andaluza de Salud Pública (EASP), Consejería de Salud y Familias de la Junta de Andalucia, Cuesta del Observatorio,4, 18080 Granada, Spain

**Keywords:** Evictions, Foreclosure, Suicide risk, Social support, Banks

## Abstract

**Background:**

One of the greatest effects of the financial crisis in Spain has been the enormous increase in the number of evictions. Several studies have shown the association of evictions with different aspects of the physical and mental health. Furthermore, evictions have been associated with an increased risk of suicide. Our objective was to evaluate the risk of suicide among victims of eviction and investigate whether it is associated with specific characteristics of households and interviewees, the eviction process and social support, and health needs.

**Methods:**

A total of 205 participants from households threatened with eviction in Granada, Spain, and 673 being the total number of members of these households, were interviewed in one-on-one sessions between April 2013 and May 2014. Through a questionnaire, information was obtained on physical and mental health, characteristics of their eviction process and support networks, and the use of health services.

**Results:**

Almost half of the sample (46.7%) were at low (11.8%), moderate (16.9%), or high suicide risk (17.9%). Household and interviewee features had a limited association with suicide risk. On the contrary, the risk of suicide is greater with a longer exposure to the eviction process. In addition, threatening phone calls from banks increased significantly the risk of suicide, especially among men. Suicide risk was also associated with low social support, especially among women. Interviewees at risk of suicide received more help from nongovernmental organizations than those who were not at risk. In interviewees at risk, the main unmet needs were emotional and psychological help, especially in men. A high percentage of those at risk of suicide declare having large unmeet health needs. Finally, there was a tendency among the evicted at risk of suicide to visit emergency room and primary care more often than those not at risk, especially among women.

**Conclusions:**

To our knowledge, this is the first study showing that when banks adopt a threatening attitude, suicide risk increases among the evicted. As hypothesized, when the evicted felt socially supported, suicide risk decreased. Emotional help was the main mediator of suicide risk and the main unmet need, especially among men.

## Backgroud

In 2007, after decades of irresponsible mortgage practices [1], the financial sector suddenly crumbled, causing the worldwide collapse of the housing market and, in turn, an unprecedented increase in home foreclosures. The impact of the crisis was heterogeneous and in Spain, it hit Mediterranean regions such as Andalusia the most [2]. As in earlier economic crises, the Great Recession resulted in worse mental health and higher suicide rates [3–13], especially in low-income, high-unemployment areas [1, 7, 10, 14–16].

Home evictions consist of a long process, from mortgage arrears to the loss of the home, during which the household is increasingly pressured to pay back the loans. In Spain during the harshest years of the crisis (2008–2013) housing-related legal actions were essentially dispossessions related to home ownership rather than renting [17] . The eviction process has been associated with stress, loss of identity, shame, and failure [18–21], which lead to poor mental health [7, 16], visible in higher rates of depression and anxiety among the evicted, and symptomatology of post-traumatic stress disorder [PTSD] [21–23].

Suicide is the worst possible outcome of disturbed mental health [24]. It has been estimated that each suicide corresponds to about 20 failed attempts [25, 26]. Studies in several countries have consistently shown that home evictions are associated with higher suicide rates, even after adjusting for unemployment, debts, and previous mental health [7, 27–29]. In most countries, the evicted population comprises socially vulnerable groups, hence, it is hard to disentangle the impact of eviction from the impact of other forms of social marginalization. However, the main cause of evictions in Spain during the economic crisis was the loss of employment, mostly affecting the working middle class, unlike in other countries, where evictions are part of a complex process of marginalization of the structurally vulnerable population [15, 22, 28, 30].

Research on economic downturns and mental health across different countries emphasizes the role of the state [31]: states that adopted drastic measures of austerity by limiting welfare, such as Spain, Portugal, and Greece, reported sharper increases in mortality and suicide rates [10, 16, 32–34], as compared to countries that rejected austerity, such as Iceland, or provided formal support by increasing social welfare, such as Sweden and Finland– [3, 34, 35]. Overall, these findings highlight the mediating role of formal support in the relationship between economic downturns and suicides.

Spain is among the European countries hardest hit by the crisis [36]. In recent decades, government policies promoted housing as a central component of the Spanish economy, while eliminating key policies of employment protection, causing a sustained increase in low wages, insecurity and job insecurity [37, 38]. The housing market crash tripled national unemployment rates, which were the highest or the second-highest in Europe-28 from 2009 to 2018 [39], and struck Spain’s working middle class [37], causing shattering levels of family poverty [40]. Furthermore, Spanish mortgage laws, criticized by 46 senior Spanish judges and denounced by the Court of Justice of the European Union [CJEU] [41], in case of arrears, do not offer alternatives to foreclosure and do not allow the mortgagor to satisfy the debt by selling/returning the property, therefore, the value of the property must still be paid after eviction. Consequently, a portion of Spain’s middle class became part of the new poor and increased the evicted population [15, 22]. In the absence of state support, families and the voluntary sector (eg, Caritas, parishes] provided material assistance, while affected citizens joined a network of non-governmental organizations to protest and provide legal and social support to families threatened of eviction. In Spain, the main support platform is Stop Evictions-Platform of Affected by Mortgage-PAH [*Stop Desahucios-Plataforma de Afectados por la Hipoteca-PAH*] [40, 42].

The risk of suicide is often used as the probability that a person commits suicide or executes some suicidal behavior. Several factors are known to increase this risk. Among them are factors associated with the household or the subject, such as demographics, socio-economic and employment conditions, family history, or mental health conditions, as well as stressful life events, such as serious economic and legal difficulties, as in an eviction process. On the other hand, social and family support are well-known protective factors against suicide [43, 44].

The present study investigated suicide risk in 205 households threatened with eviction (674 members) by conducting one-on-one interviews with attendees of Stop Evictions meetings in Granada, Spain. Granada, in the southern region of Andalusia, has high unemployment rates and among the highest suicide rates in Spain [10, 11, 45]. The goal of this study was to assess whether suicide risk was associated with specific features of the household (interviewee- or household-related), of the eviction process, or of support networks. Specifically, did suicide risk depend on the specific conditions of the individuals or their households? Did the duration of the eviction process and the attitude of the banks (including the two types of banks that grant mortgages in Spain, private commercial banks and public savings banks-cajas de ahorros) influence the suicide risk of mortgage victims? What was the role of social support: did formal, informal, and personal support networks mitigate suicide risk?

## Methods

### Context

The data belong to a wider cross-sectional study on the impact of evictions on health. A survey assessed physical and mental health, features of the eviction process and of support networks, and use of health services. Findings on self-perceived general and mental health -PTSD, depression, and anxiety symptomatology- were reported elsewhere [22, 30, 46]. The field work was conducted between April 2013 and May 2014. Trained professionals individually administered the survey during the weekly meetings of the Stop Evictions platform that took place in Granada, Spain. Immediately before the meetings, attendants currently threatened with eviction at the time were presented the study and invited to participate, and those who agreed were interviewed in the following meeting. All participants provided written informed consent and were informed that their responses were confidential. The research project was approved by the Andalusian Health System Research Ethics Committee.

### Sample

Participants were recruited among attendees of the weekly meetings of the Granada Stop Evictions platform. For the recruitment, we followed (but not strictly) the methodology called “Respondent-driven sampling”, given its suitability for hard-to-reach groups [47, 48]. The recruitment began in the moments before the weekly meetings of the assemblies, and the study was presented to the attendees and they were invited to participate. From there, subjects were recruited for several weeks. The sampling process ended when the size of the sample approached the target number initially estimated, and the number of subjects recruited was so small that it indicated the end of the subjects available or willing to participate.

The interviewees were 205 adult mortgage victims (> 18 years), *each representing a household* at any stage of the eviction process –from mortgage arrears to having been evicted–. For instance, if three adult household members attended the meeting, only one was interviewed. Individuals who attended the meetings but were not under the threat of eviction could not take part in the study.

### Variables

Our main focus was suicide risk. For this purpose the MINI International Psychiatric Interview [49] was used to identify mortgage victims showing no risk, low risk, moderate risk, or high risk of suicide. Due to the small size of the sample, the variable “risk of suicide” was dichotomized to compare victims at risk of suicide (low, moderate, high) with victims who did not show risk on that scale.

The survey, available upon request, investigated whether suicide risk was modified by the following characteristics of: 1) the household; 2) the eviction process -its duration and banks’ attitudes-; 3) social support-perceived, solicited, and received, use of health care services, unmet support and health needs.

#### Household

Household features included 1) characteristics of the interviewee: demographics sex, age, civil status, educational level, employment, occupational social class based on the 2011 Spanish classification of occupations [50], and provider role; 2) characteristics of the household: household size, current monthly income, perceived financial strain [“each month, how hard is it to make ends meet?”), and the presence of children or disabled members.

#### Eviction process

To investigate whether banks contributed to mortgage victims’ suicide risk, the survey evaluated 1) the stages and duration of the eviction –that is, whether the household was in arrears or in advanced stages of the eviction process and whether the household had been exposed recently or for more than 2 years to the threat of eviction. These variables allowed us to examine whether longer exposure to the eviction process was associated with a greater suicide risk. 2) Banks’ attitude during phone calls -whether banks adopted a threatening rather than conciliating attitude while soliciting mortgage payback.

#### Perceived support and support networks

The Duke-UNK Functional Social Support Scale [51] was employed to investigate mortgage victims’ perceived social support. The scale distinguishes subjectively high or low social support and identifies whether the individual feels supported or not rather than the actual support received.

Actual support was investigated by assessing the formal, informal, and personal social support networks available. In this study, formal support networks refer here to health care services, informal support networks refer to nongovernmental organizations such as parishes, Caritas, and the Stop Evictions platform, and social support networks consist of family and friends. For each type of support network, we investigated, if support was solicited, which support network -formal, informal, or personal- was the main support provider, the type of support received-material, emotional, or legal- and unmet support needs.

As for health care services, the following were analyzed: 1) Emergency Room [ER] visits in the last year, 2) primary care visits in the last 2 weeks and the type of health professional consulted (physician, psychologist or other), 3) unmet health care needs [“health care services you would have needed to use but could not”).

### Statistical analysis

All statistical analyses were performed using SPSS 25.0 [Chicago, IL, USA]. The characteristics of the sample were first examined using descriptive statistics. Next, using Pearson’s χ^2^ or Fisher’s exact test when appropriate, it was investigated whether the presence of suicide risk was associated to specific features of the interviewee/household, eviction process, and support networks.

## Results

### Sample characteristics

Table 1 shows the suicide risk levels and demographic, employment, and household features of the sample. The data correspond to the 205 participants in the study, representing one household each, with 673 being the total number of members of these households.

**Table 1:** Suicide risk, Demographics, employment, and household features

	Men 83 (40.5%)	Women 122 (59.9%)	Total
Suicide Risk (levels)^a^			
No risk	42 (52.5%)	62 (53.9%)	104 (53.3%)
Low risk	10 (12.5%)	13 (11.3%)	23 (11.8%)
Moderate risk	17 (21.3%)	16 (13.9%)	33 (16.9%)
High risk	11 (13.8%)	24 (10.9%)	35 (17.9%)
Total	80 (100%)	115 (100%)	195 (100%)
Age			
25 to 35 years	29 (34.9%)	29 (23.8%)	58 (28.3%)
36 to 50 years	36 (43.4%)	68 (55.7%)	104 (50.7%)
51 to 74 years	18 (21.7%)	25 (20.5%)	43 (21.0%)
Total	83 (100%)	122 (100%)	205 (100%)
Civil status			
Married	43 (54.4%)	59 (49.2%)	102 (51.3%)
Unmarried	25 (31.6%)	27 (22.5%)	52 (26.1%)
Separated/Divorced	11 (13.9%)	28 (23.3%)	39 (19.6%)
Widowed	0 (0%)	6 (5%)	6 (3.0%)
Total	79 (100%)	120 (100%)	199 (100%)
Education level			
Primary	42 (50.6%)	61 (50.8%)	103 (50.7%)
Secondary	37 (44.6%)	42 (35%)	79 (38.9%)
University	4 (4.8%)	17 (14.2%)	21 (10.3%)
Total	83 (100%)	120 (100%)	203 (100%)
Employment			
Self-employed	3 (3.7%)	4 (3.5%)	7 (3.6%)
Permanent contract	4 (4.9%)	13 (11.4%)	17 (8.7%)
Precarious employment ^b^	6 (7.4%)	21 (18.4%)	27 (13.8%)
Unemployed	61 (75.3%)	62 (54.4%)	123 (63.1%)
Retired/invalidity pension	6 (7.4%)	58 (2.6%)	64 (32.8%)
Homemaker	1 (1.2%)	11 (9.6%)	12 (6.2%)
Total	81 (100%)	114 (100%)	195 (100%)
Occupational social class^c^			
I	6 (7.5%)	5 (4.5%)	11 (5.8%)
II	33 (41.3%)	28 (25.2%)	61 (31.9%)
III	25 (31.3%)	36 (32.4%)	61 (31.9%)
IV	16 (20%)	42 (37.8%)	58 (30.4%)
Total	80 (100%)	111 (100%)	191 (100%)
Provider			
Yes	47 (57%)	59 (50%)	106 (52.7%)
No	36 (43%)	59 (50%)	95 (47.3%)
Total	83 (100%)	118 (100%)	201 (100%)
Household size			
< 2 members	23 (28%)	33 (27.3%)	56 (27.6%)
3 to 5 members	57 (69.5%)	80 (66.1%)	137 (67.5%)
> 5 members	2 (2.4%)	8 (6.6%)	10 (4.9%)
Total	82 (100%)	121 (100%)	203 (100%)
Current monthly income (euros)			
< 500	48 (45.8%)	51 (42.5%)	99 (48.8%)
501–1000	28 (33.7%)	49 (40.8%)	77 (37.9%)
> 1000	17 (20.5%)	20 (16.7%)	37 (18.2%)
Total	83 (100%)	120 (100%)	203 (100%)
Perceived financial strain (monthly)			
Hard	79 (97.5%)	110 (98.2%)	189 (97.9%)
Easy	2 (2.5%)	2 (1.8%)	4 (2.1%)
Total	81 (100%%)	112 (100%%)	193 (100%)
Children in the house			
Yes	45 (59.2%)	71 (65.1%)	116 (62.7%)
No	31 (40.8%)	38 (34.9%)	69 (37.3%)
Total	76 (100%)	109 (100%)	185 (100%)
Disabled members in the house			
Yes	12 (15%)	25 (21.2%)	37 (18.7%)
No	68 (85%)	93 (78.8%)	161 (81.3%)
Total	80 (100%)	118 (100%)	198 (100%)

^a^MINI International Psychiatric Interview [47]

^b^Precarious employment: temporary, insecure and flexible employment

^c^ Domingo-Salvany et al., 2013 [48]

The majority of interviewees were women (59.9%). Almost half of interviewees (46.7%) were at low (11.8%), moderate (16.9%), or high suicide risk (17.9%); and one-third thought about suicide (33.3%).

One third of interviewees were older than 36 years. Half of the interviewees had a primary education (50.7%), the other half had a secondary (39.8%) or university education (9.5%). Most men were unemployed (75%), whereas among women 54.4% were unemployed, 18.4% were in precarious employment, 11.4% had permanent contracts, and 9.6% were homemakers. Approximately one-third of mortgage victims belonged to the II, III, and IV occupational class and half were providers. Most households comprised more than three members. Almost half lived on less than 500 euros/month (44.15%), the rest lived on 501 to 1000 (44.15%) or more than 1000 euros/month (18.6%). Virtually all households reported difficulties making ends meet (97.85%). Most households included children (62.15%) and almost one-fifth included disabled members (18.10%).

### Household and suicide risk

Table 2 shows suicide risk as a function of the interviewee and the household features. In general, there was no specific association, however in men employment and occupational social class tended to be associated with suicide risk (both *p* < 0.05).

**Table 2:** Interviewee and household features and suicide risk

	Suicide risk											
	Men n (%)				Women n (%)				Total n (%)			
	No	Yes	Total	p	No	Yes	Total	p	No	Yes	Total	p
Age												
25 to 35 years	15 (35.7%)	12 (31.6%)	27 (33.8%)		17 (27.4%)	11 (20.8%)	28 (24.3%)		32 (30.8%)	23 (25.3%)	55 (28.2%)	
36 to 50 years	19 (45.2%)	17 (44.7%)	36 (45.0%)		34 (54.8%)	29 (54.7%)	63 (54.8%)		53 (51.0%)	46 (50.5%)	99 (50.8%)	
51 to 74 years	8 (19.0%)	9 (23.7%)	17 (21.3%)		11 (17.7%)	13 (24.5%)	24 (20.9%)		19 (18.3%)	22 (24.2%)	41 (21.0%)	
Civil status												
Married	21 (52.5%)	20 (55.6%)	41 (53.9%)		32 (52.5%)	24 (45.3%)	56 (49.1%)		53 (52.5%)	44 (49.4%)	97 (51.1%)	
Unmarried	11 (27.5%)	13 (36.1%)	24 (31.6%)		14 (23.0%)	13 (24.5%)	27 (23.7%)		25 (24.8%)	26 (29.2%)	51 (26.8%)	
Separated; divorced; widowed.	8 (20.0%)	3 (8.3%)	11 (14.5%)		15 (24.6%)	16 (30.2%)	31 (27.2%)		23 (22.8%)	19 (21.3%)	42 (22.1%)	
Education level												
Primary	23 (54.8%)	28 (73.7%)	51 (63.8%)		39 (62.9%)	29 (55.8%)	68 (59.6%)		62 (59.6%)	57 (63.3%)	119 (61.3%)	
Secondary	16 (38.1%)	9 (23.7%)	25 (31.3%)		14 (22.6%)	18 (34.6%)	32 (28.1%)		30 (28.8%)	27 (30.0%)	57 (29.4%)	
University	3 (7.1%)	1 (2.6%)	4 (5.0%)		9 (14.5%)	5 (9.6%)	14 (12.3%)		12 (11.5%)	6 (6.7%)	18 (9.3%)	
Employment												
Self-employed	2 (4.8%)	1 (2.8%)	3 (3.8%)		1 (1.7%)	3 (6.1%)	4 (3.7%)		3 (3.0%)	4 (4.7%)	7 (3.8%)	
Permanent contract	3 (7.1%)	1 (2.8%)	4 (5.1%)		6 (10.2%)	5 (10.2%)	11 (10.2%)		9 (8.9%)	6 (7.1%)	15 (8.1%)	
Temporary contract	0 (0.0%)	6 (16.7%)	6 (7.7%)		9 (15.3%)	12 (24.5%)	21 (19.4%)		9 (8.9%)	18 (21.2%)	27 (14.5%)	
Unemployed	33 (78.6%)	26 (72.2%)	59 (75.6%)		35 (59.3%)	24 (49.0%)	59 (54.6%)		68 (67.3%)	50 (58.8%)	118 (63.4%)	
Retired/invalidity pension	4 (9.5%)	1 (2.8%)	5 (6.4%)		3 (5.1%)	0 (0.0%)	3 (2.8%)		7 (6.9%)	1 (1.2%)	8 (4.3%)	
Homemaker	0 (0.0%)	1 (2.8%)	1 (1.3%)	*	5 (8.5%)	5 (10.2%)	10 (9.3%)		5 (5.0%)	6 (7.1%)	11 (5.9%)	†
Occupational social class												
I	3 (7.7%)	3 (7.9%)	6 (7.8%)		2 (3.4%)	2 (4.2%)	4 (3.8%)		5 (5.2%)	5 (5.8%)	10 (5.5%)	
II	17 (43.6%)	14 (36.8%)	31 (40.3%)		17 (29.3%)	9 (18.8%)	26 (24.5%)		34 (35.1%)	23 (26.7%)	57 (31.1%)	
III	7 (17.9%)	17 (44.7%)	24 (31.2%)		16 (27.6%)	19 (39.6%)	35 (33.0%)		23 (23.7%)	36 (41.9%)	59 (32.2%	
IV	12 (30.8%)	4 (10.5%)	16 (20.8%)	*	23 (39.7%)	18 (37.5%)	41 (38.7%)		35 (36.1%)	22 (25.6%)	57 (31.1%)	†
Provider												
Yes	22 (52.4%)	24 (63.2%)	46 (57.5%)		30 (48.4%)	25 (50.0%)	55 (49.1%)		52 (50.0%)	49 (55.7%)	101 (52.6%)	
No	20 (47.6%)	14 (36.8%)	34 (42.5%)		32 (51.6%)	25 (50.0%)	57 (50.9%)		52 (50.0%)	39 (44.3%)	91 (47.4%)	
Household size												
< 2 members	14 (33.3%)	9 (24.3%)	23 (29.1%)		13 (21.0%)	19 (35.8%)	32 (27.8%)		27 (26.0%)	28 (31.1%)	55 (28.4%)	
3 to 5 members	28 (66.7%)	26 (70.3%)	54 (68.4%)		45 (72.6%)	30 (56.6%)	75 (65.2%)		73 (70.2%)	56 (62.2%)	129 (66.5%)	
> 5 members	0 (0.0%)	2 (5.4%)	2 (2.5%)		4 (6.5%)	4 (7.5%)	8 (7.0%)		4 (3.8%)	6 (6.7%)	10 (5.2%	
Current monthly income (euros)												
< 500	21 (50.0%)	16 (42.1%)	37 (46.3%)		26 (41.9%)	22 (43.1%)	48 (42.5%)		47 (45.2%)	38 (42.7%)	85 (44.0%)	
501–1000	12 (28.6%)	14 (36.8%)	26 (32.5%)		26 (41.9%)	23 (45.1%)	49 (43.4%)		38 (36.5%)	37 (41.6%)	75 (38.9%)	
> 1000	9 (21.4%)	8 (21.1%)	17 (21.3%)		10 (16.1%)	6 (11.8%)	16 (14.2%)		19 (18.3%)	14 (15.7%)	33 (17.1%)	
Perceived financial strain (monthly)												
Hard	39 (97.5%)	37 (97.4%)	71 (97.3%)		57 (100.0%)	48 (98.0%)	105 (99.1%)		96 (99.0%)	85 (97.7%)	181 (98.4%)	
Easy	1 (2.5%)	1 (2.6%)	2 (2.7%)		0 (0%)	1 (2.0%)	1 (0.9%)		1 (1.0%)	2 (2.3%)	3 (1.6%)	
Children in the house												
Yes	23 (59.0%)	21 (60.0%)	44 (59.5%)		41 (70.7%)	26 (56.5%)	67 (64.4%)		64 (66.0%)	47 (58.0%)	111 (62.4%)	
No	16 (41.0%)	14 (40.0%)	30 (40.5%)		17 (29.3%)	20 (43.5%)	37 (35.6%)		33 (34.0%)	34 (42.0%)	67 (37.6%)	
Disabled members in the house												
Yes	6 (14.3%)	6 (17.1%)	12 (15.6%)		10 (16.7%)	14 (26.9.3%)	24 (21.4%)		16 (15.7%)	20 (23.0%)	36 (40.4%)	
No	36 (85.7%)	29 (82.9%)	65 (84.4%)		50 (83.3%)	38 (73.1%)	88 (78.6%)		86 (84.3%)	67 (77.0%)	53 (59.6%)	

†*p* < .10 *p < .05 **p < .01 ***p < .001

### Eviction process and suicide risk

As Table 3 shows, all features of the eviction process were associated with suicide risk, especially in women. Female mortgage victims, in the late stages of the eviction process, had a greater suicide risk (*p* < 0.05), whereas for men, suicide risk was similar in early and late stages of the eviction process. Similarly, the duration of the eviction process was clearly associated with suicide risk (*p* < 0.001), so that in a high percentage of men (*p* < 0.01) and women (*p* < 0.05) suicide risk increased with prolonged exposure to the eviction process. Furthermore, the attitude of banks correlated with suicide risk, so that threatening phone calls from banks were associated with a greater suicide risk (*p* < 0.05), especially in males (*p* < 0.05).

**Table 3:** Eviction process, banks’ attitude, and suicide risk

	Suicide risk											
	Men n (%)				Women n (%)				Total n (%)			
	No	Yes	Total	p	No	Yes	Total	p	No	Yes	Total	p
Stage in eviction												
In arrears	20 (50.0%)	19 (51.4%)	39 (50.6%)		43 (72.9%)	23 (50.0%)	65 (62.5%)		63 (63.6%)	42 (50.6%)	105 (57.7%)	
Ongoing eviction	20 (50.0%)	18 (48.6%)	38 (49.4%)		16 (27.1%)	23 (50.0%)	39 (37.5%)	*	36 (36.4%)	41 (49.4%)	77 (42.3%)	†
Duration of the eviction process												
Recent	32 (88.9%)	22 (62.9%)	54 (76.1%)		37 (74.0%)	26 (53.1%)	63 (63.6%)		69 (80.2%)	48 (57.1%)	117 (68.8%)	
> 2 years	4 (11.1%)	13 (37.1%)	17 (23.9%)	**	13 (26.0%)	23 (46.9%)	36 (36.4%)	*	17 (19.8%)	36 (42.9%)	53 (31.2%)	***
Banks’ attitude during phone calls												
Conciliating	15 (68.2%)	8 (36.4%)	23 (52.3%)		18 (64.3%)	18 (51.4%)	36 (57.1%)		33 (66.0%)	26 (45.6%)	59 (50.9%)	
Threatening	7 (31.8%)	14 (63.6%)	21 (47.7%)	*	10 (35.7%)	17 (48.6%)	27 (42.4%)	*	17 (34.0%)	31 (54.4%)	57 (49.1%)	*

†p < .10 *p < .05 **p < .01 ***p < .001

### Social support and suicide risk

Based on Duke’s-UNC scale, most mortgage victims (66.3%) felt high social support, 74.7% in men, and 60.7% in women.

Table 4 shows that suicide risk was associated with low social support (*p* < 0.01), especially among women (*p* < 0.01).

**Table 4:** Social support and suicide risk. Support perceived, solicited, received, and unmet support needs

	Suicide risk											
	Men n (%)				Women n (%)				Total n (%)			
	No	Yes	Total	p	No	Yes	Total	p	No	Yes	Total	p
Support perceived (Duke’s-UNC scale)												
High	33 (78.6%)	27 (71.1%)	60 (75%)		46 (74.2%)	23 (43.4%)	69 (60%)		79 (76%)	50 (54.9%)	129 (66.2%)	
Low	9 (21.4%)	11 (28.9%)	20 (25.0%)		16 (25.8%)	30 (56.6%)	46 (40%)	***	25 (24%)	41 (45.1%)	66 (33.8%)	**
Source of support solicited^a^												
Formal support networks												
No	28 (66.7%)	16 (42.1%)	44 (55%)		33 (53.2%)	20 (37.7%)	53 (46.1%)		61 (58.7%)	36 (39.6%)	97 (49.7%)	
Yes	14 (33.3%)	22 (57.9%)	36 (45.0%)	*	29 (46.8%)	33 (63.%)	62 (53.9%)	†	43 (41.3%)	55 (60.4%)	98 (50.3%)	**
Informal support networks												
No	32 (76.2%)	26 (68.4%)	58 (72.5%)		42 (67.7%)	32 (60.4%)	74 (64.3%)		74 (71.4%)	58 (63.7%)	132 (67.7%)	
Yes	10 (23.8%)	12 (31.6%)	22 (27.5%)		20 (32,3%)	21 (39.6%)	41 (35.7%)		30 (28.8%)	33 (36.3%)	63 (32.3%)	
Family/friends												
No	12 (28.6%)	20 (52.6%)	32 (40.0%)		25 (40.3%)	17 (32.1%)	42 (36.5%)		37 (35.6%)	37 (40.7%)	74 (37.9%)	
Yes	30 (71.4%)	18 (47.4%)	48 (60.0%)	*	37 (59.7%)	36 (67.9%)	73 (63.5%)		67 (64.4%)	54 (59.3%)	121 (62.1%)	
Source of support received^b^												
Formal support networks												
No	3 (21.4%)	7 (31.8%)	10 (27.8%)		14 (48.3%)	11 (33.3%)	25 (40.3%)		17 (39.5%)	18 (32.7%)	35 (35.7%)	
Yes	11 (78.6%)	15 (68.2%)	26 (72.2%)		15 (51.7%)	22 (66.7%)	37 (59.7%)		26 (60.5%)	37 (67.3%)	63 (64.3%)	
Informal support networks												
No	0 (0.0%)	1 (8.3%)	1 (4.5%)		9 (45.0%)	2 (9.5%)	11 (26.8%)		9 (30.0%)	3 (9.1%)	12 (19.0%)	
Yes	10 (100%)	11 (91.7%)	21 (95.5%)		11 (55,0%)	19 (90.5%)	30 (73.2%)	**	21 (70%)	30 (90.9%)	51 (81.0%)	*
Family/friends												
No	1 (3.3%)	2 (11.1%)	3 (6.3%)		5 (13.5%)	8 (22.2%)	13 (17.8%)		6 (9.0%)	10 (18.5%)	16 (13.2%)	
Yes	29 (96.7%)	16 (88.9%)	45 (93.8%)		32 (86.5%)	28 (77.8%)	60 (82.2%)		61 (91.0%)	44 (81.5%)	105 (86.8%)	
Type of help received, regardless the support network												
Material help												
No	14 (34.0%)	15 (37.5%)	29 (35.4%)		25 (37.9%)	14 (25.5%)	39 (32.2%)		39 (36.1%)	29 (30.5%)	68 (33.5%)	
Yes	28 (66.7%)	25 (62.5%)	53 (64.6%)		41 (62.1%)	41 (74.5%)	82 (67.8%)		69 (63.9%)	66 (69.5%)	135 (66.5%)	
Emotional help												
No	16 (38.1%)	28 (70.0%)	44 (53.7%)		32 (48.5%)	24 (44.4%)	56 (46.7%)		48 (44.4%)	52 (55.3%)	100 (49.5%)	
Yes	26 (61.9%)	12 (30.0%)	38 (46.3%)	**	34 (51.5%)	30 (55.6%)	64 (53.3%)		60 (55.6%)	42 (44.7%)	102 (50.5%)	
Legal help												
No	33 (79.6%)	33 (82.5%)	66 (80.5%)		48 (72.7%)	47 (87.0%)	95 (79.2%)		81 (75.0%)	80 (85.1%)	161 (79.7%)	
Yes	9 (21.4%)	7 (17.5%)	16 (19.5%)		18 (27.3%)	7 (13.0%)	25 (20.8%)		27 (25.0%)	14 (14.9%)	41 (20.3%)	
Amount of unmet support needs (up to 3)												
None	1 (2.4%)	0 (0%)	1 (1.3%)		8 (12.9%)	4 (7.5%)	12 (10.4%)		9 (8.7%)	4 (4.4%)	13 (6.7%)	
1	19 (45.2%)	14 (36.8%)	33 (41.3%)		26 (41.9%)	14 (26.4%)	40 (34.8%)		45 (43.3%)	28 (30.8%)	73 (37.4%)	
2	14 (33.3%)	10 (26.3%)	24 (30.0%)		22 (35.5%)	14 (26.4%)	36 (31.3%)		36 (34.6%)	24 (26.4%)	60 (30.8%)	
3	8 (19.0%)	14 (36.8%)	22 (27.5%)		6 (9.7%)	21 (39.6%)	27 (23.5%)	**	14 (13.5%)	35 (38.5%)	49 (25.1%)	***
Type of unmet support needs.												
Seeking an alternative house												
Not selected	37 (88.1%)	33 (86.8%)	70 (87.5%)		59 (95.2%)	47 (88.7%)	106 (92.2%)		96 (92.3%)	80 (87.9%)	176 (90.3%)	
Selected	5 (11.9%)	5 (13.2%)	10 (12.5%)		3 (4.8%)	6 (11.3%)	9 (7.8%)		8 (7.7%)	11 (12.1%)	19 (9.7%)	
Psychological help												
Not selected	32 (57.9%)	24 (42.1%)	54 (100%)		45 (72.5%)	28 (52.8%)	73 (63.5%)		77 (74.0%)	50 (54.9%)	127 (65.1%)	
Selected	10 (23.8%)	16 (64.0%)	26 (32,5%)	†	17 (27.4%)	25 (47.2%)	42 (36.5%)	*	27 (26.0%)	41 (59.4%)	68 (34.6%)	**
Emotional help												
Not selected	34 (81.0%)	24 (63.2%)	58 (72.5%)		49 (79.0%)	29 (54.7%)	78 (67.8%)		83 (79.8%)	53 (58.2%)	136 (69.7%)	
Selected	8 (19.0%)	14 (36.8%)	22 (27.5%)	†	13 (21.0%)	24 (45.3%)	37 (32.2%)	**	21 (20.2%)	38 (41.8%)	59 (30.3%)	***
Better health and social care support												
No	36 (85.7%)	32 (84.2%)	68 (85.0%)		57 (91.9%)	46 (86.8%)	103 (89.6%)		80 (59.7%)	54 (40.3%)	134 (84.8%)	
Yes	6 (14.3%)	6 (15.8%)	12 (15.0%)		5 (8.1%)	7 (13.2%)	12 (10.4%)		11 (10.6%)	13 (14.3%)	24 (12.3%)	
Legal help												
No	26 (61.9%)	26 (68.1%)	52 (65.0%)		47 (75.8%)	40 (75.5%)	87 (75.7%)		73 (70.2%)	66 (72.5%)	139 (71.3%)	
Yes	16 (38.1%)	12 (31.6%)	28 (35.0%)		15 (24.2%)	13 (24.5%)	28 (24.3%)		31 (29.8%)	25 (27.5%)	56 (28.7%)	
Negotiation with banks												
No	22 (52.4%)	21 (55.3%)	43 (53.8%)		38 (61.3%)	30 (56.6%)	68 (59.1%)		60 (57.7%)	51 (56.0%)	111 (56.9%)	
Yes	20 (47.6%)	17 (44.7%)	37 (46.3%)		24 (38.7%)	23 (43.4%)	47 (40.9%)		44 (42.3%)	40 (44.0%)	84 (43.1%)	

†p < .10 *p < .05 **p < .01 ***p < .001

^a^‘To whom did you solicit support?’

^b^‘Among solicited support networks, from whom did you receive support?’

Mortgage victims solicited support especially to family and friends (62.1%), to formal support networks, i.e., social and health care (50.3%), and to a lesser extent to informal support networks such as nongovernmental organizations (32.3%). When at risk of suicide, interviewees solicited mostly formal support (*p* < 0.01), especially if they were men (*p* > 0.05). Consistently, men at risk of suicide solicited less help from families and friends (47.4%) than men who were not at risk of suicide (71.4%). As for the actual support received, family and friends were the main source of help when the evicted solicited it (86.8%). Interviewees at risk of suicide received more help from nongovernmental organizations than those who were not at risk (81%, *p* < 0.05). There was no difference in the amount of formal support received between those at risk of suicide and those without risk.

Regardless of the source of support, interviewees received mostly material, i.e., money, food (66.5%), and emotional help (50.5%). Only emotional help was associated with suicide risk in men (*p* < 0.01): 70% of men at risk reported insufficient emotional help. As for unmet support needs, interviewees at risk disclosed a greater amount of unmet needs (*p* < 0.001), especially if they were women (*p* < 0.01). The main unmet need was support in mediating with banks (43.1%), followed by psychological, emotional, and legal help. In interviewees at risk, the main unmet needs were emotional (*p* < 0.001) and psychological help (*p* < 0.01), especially in men (*p* < 0.01 and *p* < 0.05, respectively).

Table 5 describes the use of health care services, unmet health care needs, and their interaction with mortgage victims’ suicide risk.

**Table 5:** Use of health care services and unmet health needs

	Suicide risk											
	Men n (%)				Women n (%)				Total n (%)			
	No	Yes	Total	p	No	Yes	Total	p	No	Yes	Total	p
Use of health care services												
ER visits (last year)												
No	24 (57.1%)	19 (50.0%)	43 (53.8%)		30 (49.2%)	17 (32.7%)	47 (41.6%)		54 (52.4%)	36 (40.0%)	90 (46.6%)	
Yes	18 (42.9%)	19 (50.0%)	37 (46.3%)		31 (50.8%)	35 (67.3%)	66 (58.4%)	†	49 (47.6%)	54 (60.0%)	103 (53.4%)	†
Primary care visits (last 2 weeks)												
No	31 (73.8%)	24 (66.7%)	55 (70.5%)		39 (63.9%)	25 (48.1%)	64 (56.6%)		70 (68.0%)	49 (55.7%)	119 (62.3%)	
Yes	11 (26.2%)	12 (33.3%)	23 (29.5%)		22 (36.1%)	27 (51.9%)	49 (43.4%)	†	33 (32.0%)	39 (44.3%)	72 (37.7%)	†
Type of health professional consulted (last 2 weeks)												
Physician	9 (81.8%)	8 (66.7%)	17 (73.9%)		14 (63.6%)	19 (70.4%)	33 (67.3%)		23 (69.7%)	27 (69.2%)	50 (69.4%)	
Psychologist	1 (9.1%)	3 (25%)	4 (17.4%)		0 (0.0%)	1 (3.7%)	1 (2.0%)		1 (3.0%)	4 (10.3%)	5 (6.9%)	
Other	1 (9.1%)	1 (8.3%)	2 (8.7%)		8 (36.4%)	7 (25.9)	15 (30.6%)		9 (27.3%)	8 (20.5%)	17 (23.6%)	
Unmet health care needs^a^												
Physician												
No	26 (61.9%)	13 (35.1%)	39 (49.4%)		18 (29.0%)	13 (25.0%)	31 (27.2%)		44 (42.3%)	26 (29.2%)	70 (36.3%)	
Yes	16 (38.1%)	24 (64.9%)	40 (50.6%)	*	44 (71.0%)	39 (75.0%)	83 (72.8%)		60 (57.7%)	63 (70.2%)	123 (63.7%)	†
Mental health												
No	23 (54.8%)	11 (28.9%)	34 (42.5%)		20 (32.3%)	13 (24.5%)	33 (28.7%)		43 (41.3%)	24 (26.4%)	67 (34.4%)	
Yes	19 (45.2%)	27 (71.1%)	46 (57.5%)	*	42 (67.7%)	40 (75.5%)	82 (71.3%)		61 (58.7%)	67 (73.6%)	128 (65.6%)	*

†*p* < 10 *p < .05 **p < .01 ***p < .001

^a^‘Which health care services would you have liked to use, but did not?’

There was a tendency for the evicted at risk of suicide to visit ER and primary care more often than those not at risk (*p* < 0.10), especially among women (*p* < 0.10). Regardless suicide risk, half of mortgage victims (53.4%) visited the ER during the past year and almost 40% visited a health professional in the past 2 weeks. Among individuals at risk who visited a health professional, most visited a physician (69.4%), and only 6.9% visited a psychologist. As for unmet health needs, almost two-thirds of male interviewees at risk of suicide would have liked to visit a physician (p < 0.05) or a mental health professional (p < 0.05) but did not.

## Discussion

The data suggest that suicide risk was higher among the evicted: almost 50% of mortgage victims (46.7%) had a low (11.8%), moderate (16.9%), or high suicide risk (17.9%). A European study (COURAGE) estimated the prevalence of suicide ideation, planning, and intent among adult Spaniards at 3.7, 1.9, and 1.5% [52]. Another study (ESEMED) likewise estimated the Spanish prevalence of suicide ideas and attempts at 4.4 and 1.6% [53]. Naturally, it is problematic to compare data collected at different times using different measures, however, suicide risk appears clearly more frequently among mortgage victims. A Swedish study similarly observed that the risk of suicide was four times more frequent among the evicted [28].

Specifically, the present study investigated whether the risk of suicide among mortgage victims was associated with specific characteristics of the household, the interviewee, the eviction process and the support networks. The main findings were the following: household/interviewee features were scarcely associated to suicide risk, and only among men, worse employment conditions and lower social class increased suicide risk. This finding can be interpreted in the context of Spain gender inequalities and, specifically, the social expectations of men being the breadwinners [54]. When banks adopted a threatening attitude, the risk of suicide increased among mortgage victims. To our knowledge, this is the first study documenting the role of banks in mortgage victims’ suicide risk. As for social support, it was inversely related to suicide risk, being less frequent when the evicted felt socially supported. Findings on solicited, received, and unmet support and health needs pinpoint the specific lacks and needs of the evicted. Mortgage victims at risk of suicide solicited help especially from family/friends and formal networks but received it more frequently from family and nongovernmental organizations. For mortgage victims as a whole, family and friends constituted the main source of help actually received, in line with other studies showing that social networks provided around 80% of long-term care [55]. The main unmet support needed among mortgage victims was the negotiation with banks. However, people in the process of eviction who were at risk of suicide showed greater unmet needs for emotional and psychological help, than those who were not at risk of suicide. The use of health care services was higher among mortgage victims compared to the general population [56], confirming that mortgage victims searched for help in the formal healthcare system. Nonetheless, in case of suicide risk, more than two-thirds of the evicted visited a physician and identified visits to physicians or mental health professionals as the main unmet health needs.

### The role of social support

In recent decades, psychophysiological research has found a consistent relationship between social support -and its reverse, loneliness- and health: strong social support benefits the cardiovascular, endocrine, and immune systems, whereas loneliness and social isolation are associated with “greater mortality than well-established risk factors, such as cigarette smoking” [57]. Social isolation is accompanied by greater suicide risk, in adults [58–61], as well as in older men exposed to the recent economic crisis [62]. Moreover, suicide risk increases when individuals feel lonely [50, 60, 61]. Emotional support has been identified as a key dimension of social support [57], and in the current study it emerged as a key risk factor of suicide: suicide risk was less frequent when mortgage victims felt that they received sufficient emotional help, and mortgage victims at suicide risk identified emotional and psychological help as the main unmet needs.

### The role of primary care

Several professional associations have advocated the need for a formal network of support to the evicted [63–65]. In addition, attention has been repeatedly drawn to the possible very negative effects on the health of victims of evictions, suggesting the organization of community health programs at the level of primary care and considering that this level of health care is capable of managing and organizing effective interventions on mental health [66–68].

Previous publications from the World Health Organization [WHO] confirm this, further adding that “shifting mental health care to primary health care level also helps to reduce stigma, improves early detection and treatment, leads to cost efficiency and savings, and partly offsets limitations of mental health resources through the use of community resources” [69, 70]. The current findings indicate that the evicted sought help in the health care system and visited health professionals more often than the general population, overall confirming that primary care constitutes the environment most suitable for providing formal support to the evicted. In addition, this study shows that mortgage victims need more psychological help, and that they were receptive to the help they received. Therefore, it can be assumed that support networks at the primary care level could be cost effective, while generating significant benefits for the wellbeing of mortgage victims.

### The role of banks

Based on our results, banks have a clear role in mortgage victims’ suicide risk: suicide risk increases with longer exposure to the eviction process and when banks adopt a threatening attitude while soliciting payback. The possible relationship between human rights violations and health was first proposed in the mid-1980s [71] and has successively been substantiated by data showing that social injustice and inequalities prompt worse health [72, 73].

The recent economic crisis and the policies adopted to stabilize and improve the economy have worsened socioeconomic inequalities. [15, 74, 75]. And inequalities have been associated with a higher suicide risk [4, 16].

The lack of fairness in Spanish mortgage laws has been the focus of law practitioners and academics that have advocated the need of substantial changes in mortgage laws which caused a “social drama” and constitute “a legal problem” [76, 77]. While shared ownership and temporal ownership, recently studied in Spain, have been identified as feasible alternatives in case of arrears to circumvent the rigidity of mortgage laws [78], our data indicate that the main unmet need among the evicted was help in negotiating with banks, again emphasizing the lack of formal social support that Spanish mortgage victims experienced.

### Dismissal of evidence on evictions and suicide

Nonetheless, a recent 257-page-long Spanish report on “Economic crisis and health” commissioned by the Ministry of Health [79] did not mention at all, evictions or foreclosures at all, nor Spanish or international studies that describe their detrimental impact on mortgage victims’ health.

In Spain, despite the call to action for the evicted from primary health care and mental health care practitioners [64, 65] and despite the consistency of national and international scientific literature in indicating that evictions are accompanied by depression, anxiety, and a higher suicide risk [5, 10, 28, 80], the focus still appears to be on the limited reliability of the data and on the limited evidence about the impact of the crisis on the general population [79].

Accordingly, although in the report commissioned by the Spanish Ministry of Health it was acknowledged that the crisis prompted a clear worsening in mental health and in social inequities that hit disadvantaged groups the most, evictions were not mentioned, and the impact of the crisis was substantially downplayed: “the crisis [...] does not seem to have influenced health [...], apart from mental health”. The attitude toward the impact of the crisis on health has been similar in Greece: after being struck the hardest by the crisis and adopting strict austerity measures [3], findings on the adverse health consequences of the crisis were regarded as scarcely reliable and controversial [see, e.g., 82]. Such tendency to dismiss the available evidence has been described as denialism [81] and has the crucial consequence of changing priorities in state policies [38, 82]. Thus, the financial system is favored over citizens’ wellbeing, although there is reliable evidence that the worsening of mental health and the increase in suicides can be prevented or at least limited [35, 83].

### Methodological limitations and strengths

The limitations of this study are due to the cross-sectional design, the limited sample size, and the focus on mortgage victims belonging to the Stop Evictions platform. However, this platform is the only movement in Granada and most cities of Spain that offers mutual and legal support to people affected by a foreclosure process. For participant recruitment, respondent-driven sampling was used, albeit not strictly, for its suitability for hard-to-reach groups [48]. Moreover, sample characteristics were consistent with those observed in other Spanish studies on foreclosures [84, 85].

Over the 13 months of this research field work, there were about 250 foreclosures active in the courts of Granada, suggesting that the number of households included in this study (205) was adequate for the total amount of ongoing evictions [86].

In previous studies, data on suicides and evictions were mostly collected from forensic medicine registers, statistical institutions, and national databases, which render it methodologically challenging to identify and access single cases [87, 88], also due to the social stigma associated to both [19, 89]. However, the current study stems from our interest in investigating, beyond the association between evictions and suicide, the factors involved in the eviction process that might mitigate or increase mortgage victims’ suicide risk.

In a study with a similar goal [28], 22,000 Swedish households were interviewed and reported that suicide risk increased by four as a result of evictions “*independently of well-known suicidogenic risk factors preceding eviction*”. The data could be collected because the Swedish government adopted a policy of full disclosure. In Spain, the Bank of Spain -not the government- made data on home evictions available only 5 years after the crisis [78]. The link between evictions and suicides could not be investigated like in Sweden, by directly assessing all evicted households, so it was investigated by indirectly studying suicide trends in the general population [e.g., 10] or by directly accessing reduced samples like the current [22], where participants had overcome social stigma [87, 88] and to engage with mortgage victims groups. It can be speculated that at least part of the evicted who did not reach out for mortgage victims groups might have constituted worse cases, characterized by more severe social withdrawal. Therefore, the current findings might underrepresent mortgage victims’ mental health.

## Conclusions

Despite its limitations, this study helps confirm the profound psychological distress suffered by people who are in an eviction process. The risk of suicide is the “tip of an iceberg” that points to a diffused and profound experience of mental discomfort and helplessness associated with eviction, and this worsens over time. It also helps to identify some situations that aggravate this situation, such as the lack of social support and psychological and emotional help, and the menacing contact of the banks.

As concluded by Rojas and Sternberg [28] “the legitimacy of using evictions as a general preventive measure to promote [loan payback] needs to be viewed in relation to” the repercussions of evictions on mortgage victims’ health.

## Data Availability

The datasets used during the current study are available from the corresponding author on reasonable request.

## Abbreviations

CJEU: Court of Justice of the European Union
ER: Emergency room
MINI: The Mini International Neuropsychiatric Interview
PTSD: Post-traumatic stress disorder
WHO: World Health Organization
